# How to attract health workers to rural areas? Findings from a Discrete Choice Experiment in India

**DOI:** 10.1186/1753-6561-6-S5-O1

**Published:** 2012-09-28

**Authors:** Krishna D Rao

**Affiliations:** 1 Public Health Foundation of India, New Delhi, India

## Introduction

India’s success in attaining universal health coverage will critically depend on its health system’s ability to deliver clinical services in rural areas. So far, attempts to increase rural recruitment of doctors through incentives or compulsion have been unsuccessful. In 2010, we conducted a Discrete Choice Experiment (DCE) in the states of Uttarakhand and Andhra Pradesh to understand what health departments could do to make rural service attractive for doctors and nurses. Specifically, we wanted to: (a) examine the effect of monetary and non-monetary incentives on job choices and (b) develop incentive ‘packages’ with a focus on jobs in rural areas.

## Methods

Our study sample included 293 medical and nursing students and 434 in-service doctors and nurses at Primary Health Centres. An initial qualitative study identified eight job attributes i.e. health centre type, area, health facility infrastructure, staff and workload, salary, guaranteed transfer to city/town after some years of service, professional development and jobs in native area. Respondents were required to choose between a series of hypothetical job pairs that were characterized by different attribute-level combinations. Bivariate probit and mixed logistic regression analytical tools were used for the statistical analysis of the choice responses.

## Results

Results of the study suggest that individual monetary and non-monetary incentives had little effect on the uptake of rural jobs by medical students (with the exception of post-graduate seat reservation in lieu of rural service). In contrast, nursing students were more inclined towards rural jobs. Further, medical and nursing students from rural areas had a greater inclination to take up rural jobs. For in-service doctors and nurses, salary emerged as the most powerful driver of job choice. Overall, better salary, good facility infrastructure and reserving seats for higher education appeared to be the most effective drivers of uptake of rural posts for students and in-service workers. Combining these incentives can substantially increase rural recruitment.

## Discussion

In the context of India, it appears that the supply of medical graduates for rural posts is inelastic. Reserving post-graduate seats for medical graduates appears to be the strongest incentive available. In contrast, the supply is less inelastic for nursing students, in-service doctors and nurses. For them, better salary, good facility infrastructure and reserving seats for higher education appear to be the most effective drivers of uptake of rural posts. Common interventions to improve the attractiveness of rural service such as providing better housing, while important, do not appear to be the main drivers of health workers’ job choice. The study results suggest that there needs to be a focus on ‘incentive packages’ rather than the current practice of single incentives to improve recruitment in rural areas. Further, increasing the enrolment of medical and nursing students from rural backgrounds could lead to greater rural recruitment.

## Competing interests

None declared.

## Funding statement

The study was funded by the World Bank.

